# Novel transcutaneous sensor combining optical tcPO_2_ and electrochemical tcPCO_2_ monitoring with reflectance pulse oximetry

**DOI:** 10.1007/s11517-019-02067-x

**Published:** 2019-11-18

**Authors:** Willem van Weteringen, Tom G. Goos, Tanja van Essen, Christoph Ellenberger, Josef Hayoz, Rogier C. J. de Jonge, Irwin K. M. Reiss, Peter M. Schumacher

**Affiliations:** 1 Department of Pediatric Surgery, Erasmus MC - Sophia Children’s Hospital, University Medical Center Rotterdam, Rotterdam, The Netherlands; 2 Division of Neonatology, Department of Pediatrics, Erasmus MC - Sophia Children’s Hospital, University Medical Center Rotterdam, Rotterdam, The Netherlands; 3 Department of Biomechanical Engineering, Faculty of Mechanical Engineering, Delft University of Technology, Delft, The Netherlands; 4 SenTec AG, Therwil, Switzerland; 5 Pediatric Intensive Care Unit, Departments of Pediatrics and Pediatric Surgery, Erasmus MC - Sophia Children’s Hospital, University Medical Center Rotterdam, Rotterdam, The Netherlands

**Keywords:** Transcutaneous, tcPO_2_, tcPCO_2_, Oxygen, Fluorescence quenching

## Abstract

This study investigated the accuracy, drift, and clinical usefulness of a new optical transcutaneous oxygen tension (tcPO_2_) measuring technique, combined with a conventional electrochemical transcutaneous carbon dioxide (tcPCO_2_) measurement and reflectance pulse oximetry in the novel transcutaneous OxiVenT™ Sensor. In vitro gas studies were performed to measure accuracy and drift of tcPO_2_ and tcPCO_2_. Clinical usefulness for tcPO_2_ and tcPCO_2_ monitoring was assessed in neonates. In healthy adult volunteers, measured oxygen saturation values (SpO_2_) were compared with arterially sampled oxygen saturation values (SaO_2_) during controlled hypoxemia. In vitro correlation and agreement with gas mixtures of tcPO_2_ (*r* = 0.999, bias 3.0 mm Hg, limits of agreement − 6.6 to 4.9 mm Hg) and tcPCO_2_ (*r* = 0.999, bias 0.8 mm Hg, limits of agreement − 0.7 to 2.2 mm Hg) were excellent. In vitro drift was negligible for tcPO_2_ (0.30 (0.63 SD) mm Hg/24 h) and highly acceptable for tcPCO_2_ (− 2.53 (1.04 SD) mm Hg/12 h). Clinical use in neonates showed good usability and feasibility. SpO_2_-SaO_2_ correlation (*r* = 0.979) and agreement (bias 0.13%, limits of agreement − 3.95 to 4.21%) in healthy adult volunteers were excellent. The investigated combined tcPO_2_, tcPCO_2_, and SpO_2_ sensor with a new oxygen fluorescence quenching technique is clinically usable and provides good overall accuracy and negligible tcPO_2_ drift. Accurate and low-drift tcPO_2_ monitoring offers improved measurement validity for long-term monitoring of blood and tissue oxygenation.

**Figure:**
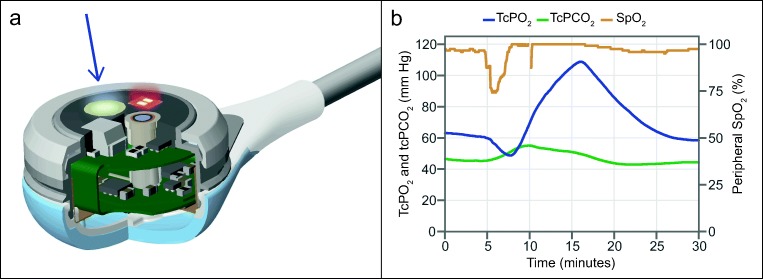
Graphical abstract

Graphical abstract

## Introduction

Transcutaneous blood gas monitoring is based on the diffusion of oxygen (O_2_) and carbon dioxide (CO_2_) from the blood to the skin surface [1]. Transcutaneous blood gas sensors locally heat the skin to induce vasodilation, resulting in an increase in supplied O_2_ and clearance of CO_2_ [2, 3]. The diffusion capacity of the skin is however markedly lower for O_2_ than for CO_2_ [4], additionally influenced by the thickness [5, 6] and microcirculatory condition [7] of the skin. As a consequence the measurement of transcutaneous oxygen (tcPO_2_) [8] requires relatively high sensor temperatures of 43 to 44 °C [9] for tcPO_2_ to correlate with arterial oxygen tension (PaO_2_), which due to skin thickness only results in tcPO_2_ values approaching PaO_2_ in infants and young children [10–12].

Conventional transcutaneous blood gas sensors are based on the electrochemical techniques introduced by Clark [13] for tcPO_2_ and Stow-Severinghaus [14] for tcPCO_2_. For decades the Clark-type electrode has been the only clinically available technique for tcPO_2_ measurements [15]. It measures oxygen by reduction, lowering the actual and thereby measured oxygen level in the superficial skin [16, 17]. Additionally there is measurement drift over time with both techniques [18], hindering usability due to reduced accuracy, frequent calibrations and membrane changes. These limitations in reliability and usability of tcPO_2_ measurements [19] have held back a widespread clinical use similar to that of tcPCO_2_ measurements. However, tcPO_2_ offers advantages over SpO_2_ in infants in which blood gas sampling is indicated for the measurement of PaO_2_, precise PaO_2_ targeting is required or the oxygen dissociation curve is markedly shifted [20, 21]. In adults the use of tcPO_2_ is limited to oxygen trend monitoring due to an insuperable underestimation of PaO_2_ [22]. In addition measurement drift hinders clinical usability. Removing measurement drift as an influence on the measurement by implementing drift-free optical techniques could therefore significantly improve usability of tcPO_2_ measurements [23]. The recently introduced OxiVenT™ Sensor (SenTec AG, Therwil, Switzerland) combines reflectance pulse oximetry and a conventional electrochemical Stow-Severinghaus-type tcPCO_2_ measurement with an optical oxygen sensing technique for measuring tcPO_2_. Fluorescence quenching [24] is the optical technique used for the measurement of oxygen, making it potentially free of drift. The main challenge in the development of this sensor was to combine two optical techniques, fluorescence quenching and pulse oximetry, without mutual interference into a single sensor which also contains an electrochemical Stow-Severinghaus tcPCO_2_ measurement. In this article we will discuss the technical aspects of implementing fluorescence quenching in a combined sensor, provide the first results on measurement accuracy and evaluate its clinical implications.

## Methods

### A novel combined transcutaneous sensor

**Fig. 1:**
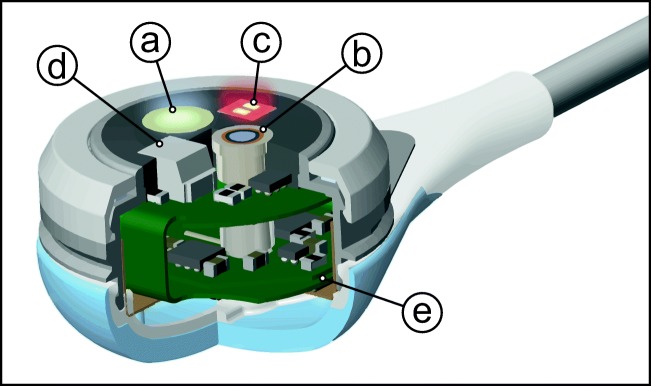
Inside view of the fully digital OxiVenT™ Sensor, showing (a) tcPO_2_ optical module, (b) tcPCO_2_ electrochemical module, (c) pulse oximetry light-emitting diode, (d) pulse oximetry photodiode, and (e) flexible circuit board containing the temperature sensors and all electronic components and microprocessor

The OxiVenT™ Sensor is the first transcutaneous sensor in which an optical tcPO_2_ measurement is combined with an electrochemical Stow-Severinghaus-type tcPCO_2_ measurement and reflective pulse oximetry (Fig. 1). The sensor weighs 2.7 g and has a diameter of 14 mm and a height of 9 mm. All measurements are digitized within the sensor and preprocessed. The principle of an electrolyte-filled diffusion chamber is retained for the tcPCO_2_ measurement. For measuring oxygen, the sensor contains an oxygen fluorescence quenching dye surface which is back-lit by an excitation light-emitting diode. On the same side of the dye, the excitation light is measured with a wavelength-filtered photodetector. In order to provide parallel optical measurements of tcPO_2_ and SpO_2_, the respective light sources emit in an alternating intermittent fashion. The sensor contains dual temperature sensing for accurate heating control. The sensor can be attached to the skin using either an ear clip or adhesive rings, minimizing pressure on the skin.

### Measuring principles and technology

#### TcPO_2_ measurement and fluorescence quenching

The OxiVenT™ Sensor measures oxygen levels with an optical technique called oxygen fluorescence quenching [24]. This technique relies on the excitation of a dye molecule by the absorption of a photon emitted by a light-emitting diode with a peak wavelength of approximately 500 nm, moving the molecule to a higher energy state. Without the presence of an oxygen molecule, the dye molecule will emit a photon at a lower specific emission wavelength (approximately 650 nm) and return to its base energy state. In the presence of an oxygen molecule, the oxygen will quench the dye and thereby prevent photon emission. In the sensing dye surface of the OxiVenT™ Sensor, fluorescence emission of each dye molecule occurs non-synchronously during a certain time interval. This results in a fluorescence intensity and decay time interval that relates to the amount of oxygen that quenches dye fluorescence. Selectively and intermittently the light intensity at the 650-nm band is measured, out of which the decay curve is reconstructed and the measured oxygen values are inferred. The oxygen diffusion to the dye results in a typical 90% response time of under 150 s. Contrary to a Clark-type electrode which reduces oxygen, influencing the oxygen level measurement itself, the fluorescence quenching technique does not affect oxygen levels.

#### TcPCO_2_ measurement

In the OxiVenT™ Sensor, CO_2_ is measured with a Stow-Severinghaus-type electrode. This technique is used in the majority of currently commercially available transcutaneous sensors and consists of a pH electrode in an electrolyte buffer containing sodium bicarbonate, covered by a gas-permeable membrane. Carbon dioxide diffuses from the skin through the membrane, where it causes a carbonic acid dissociation reaction. This in turn changes the pH of the solution, which is detected by the pH electrode and causes a potential change between the pH electrode and the reference silver/silver chloride electrode. In sensors with an electrochemical tcPO_2_ (Clark-type) and tcPCO_2_ measurement the Clark-type electrode and its inherent oxygen consumption influence pH within the diffusion chamber. Without this influence on the tcPCO_2_ measurement, there is potentially a reduction in measurement drift. Multiple patient factors and sensor temperature influence the speed at which CO_2_ diffuses from the skin, and thereby the delay in measuring the changes in arterial values transcutaneously. In practice, this delay is usually 20–80 s from changes in ventilation to their effect on transcutaneous measurements [25, 26].

#### Reflective two-wavelength pulse oximetry

In pulse oximetry, the optically measured ratio between oxygenated and deoxygenated hemoglobin is used to measure oxygen saturation. By sending two light frequencies (660-nm and 880–890-nm wavelengths) through tissue, the light intensity that results after absorption of light by the two forms of hemoglobin can be used to calculate a ratio between the two. Only the pulsatile part of the signal is analyzed as it ideally represents the arterial component of the signal. Using a calibration model, based on measurements in healthy volunteers, for each ratio, this results in a specific oxygen saturation. Although a shift in the oxygen dissociation curve can influence the interpretation of SpO_2_ values in relation to the actual PaO_2_, this technique is one of the most used oxygen monitoring techniques. Two variants of the technique are often used; transmission and reflectance pulse oximetry. In transmission pulse oximetry the light emitter and detector are placed opposite to each other on both sides of tissue (e.g. a finger), while in reflectance pulse oximetry the emitter and detector are placed next to each other. This means that in transmission pulse oximetry the light path is linear and a relatively large part of the emitted light reaches the detector. In reflectance pulse oximetry the detected light is the part that is scattered and reflected back from the tissue, resulting in a weaker signal when compared with transmission pulse oximetry. In transcutaneous sensors the arterialization caused by locally heating the skin markedly improves the reflective signal-to-noise ratio [27].

### Sensor validation methods

#### Hardware and software

All studies were performed using OxiVenT™ sensors with software versions 01.09-01.58, connected to a SenTec Digital Monitor (SDM) with software versions 08.00.0-08.01.1 (SenTec Monitoring Board) and 06.00.01-06.01.00 (Multi Parameter Board).

#### In vitro gas studies for the validation of tcPO_2_ and tcPCO_2_

An in vitro validation of the transcutaneous (O_2_ and CO_2_) measurements of the OxiVenT™ Sensor was performed with 10 sensors for each parameter in order to determine the accuracy and drift of these measurements. Prior to the protocol, the sensors were allowed to stabilize. Testing methods were in concordance with the FDA Guidance on cutaneous carbon dioxide and oxygen monitors (clause 6.2), as well as IEC 60601-2-23 [28]. Accuracy was tested by cycling through different combinations of gas concentrations of O_2_ and CO_2_. Each gas mixture was allowed to stabilize for 10 min, after which a data point was collected for each step. In the tcPCO_2_ accuracy test, a total of 4 data points for both 3% CO_2_ and 5% CO_2_ as well as 8 data points for 10% CO_2_ were collected. After 4 cycles, an additional measurement of nitrogen with 0% CO_2_ was performed. A comparable method was used for the tcPO_2_ accuracy test. This results in 4 data points for both 2% O_2_ and 10% O_2_ as well as 8 data points for 20% O_2_ after 4 cycles. Following these 4 cycles, additional measurements with nitrogen (0% O_2_) and with 100% O_2_ were performed. For the drift test, the sensors were exposed to humidified test gas (20% O_2_/10% CO_2_) for the duration of the calibration interval (24 h for tcPO_2_ and 12 h for tcPCO_2_). The total drift over the calibration interval is given as a percentage of the initial reading. In addition, the drift is given as %/h for the first hour (0–1 h) and last hour (11–12 h/23–24 h) of the calibration interval.

#### Clinical use of tcPO_2_ and tcPCO_2_

At the Neonatal Intensive Care Unit at Erasmus MC – Sophia Children’s Hospital (Rotterdam, the Netherlands), transcutaneous blood gas monitoring in preterm (24–32 weeks GA) and term neonates is performed as standard care. Existing, local, age-specific protocols for sensor temperatures and site times were applied for extreme preterm neonates (< 26 weeks GA: 42 °C, 2 h) and less preterm and term neonates (≥ 26 week GA: 43 °C, 3 h). TcPCO_2_ was calibrated initially, and when the site time elapsed, tcPO_2_ was calibrated initially and daily for verification during a tcPCO_2_ calibration. Several clinical examples were selected to demonstrate the usability and feasibility of transcutaneous blood gas monitoring of tcPO_2_ and tcPCO_2_ with the OxiVenT™ Sensor during various clinical events. SpO_2_ measurements (Masimo SET®, Masimo, Irvine, CA, USA) were recorded simultaneously with averaging over 12 s.

#### Validation of SpO_2_ in healthy volunteers

Validation of the OxiVent™ Sensor SpO_2_ measurements was performed with a clinical study in healthy volunteers at the University of California (San Francisco, USA). Approval from the institutional IRB was obtained for the study protocol. The study was carried out according to the FDA Guidance on the validation of SpO_2_ accuracy [29] and ISO 80601-2-61 [30]. The healthy volunteers underwent a desaturation protocol consisting of stepwise adjustments of the fraction of inspired oxygen (FiO_2_), targeting specific arterial oxygen saturation (SaO_2_) level plateaus. A total of two “runs” per volunteer were performed. Every SpO_2_ plateau was held for about 30–60 s. Two blood samples were collected during the saturation plateaus. Each run was then ended by several breaths of 100% O_2_ followed by room air while taking another sample pair of blood samples. The number of plateaus per “run” was adapted to the tolerance of the subjects to the desaturation protocol. SpO_2_ was measured with several sensors on 5 different application sites; *earlobe*, *forehead*, *cheek*, *upper arm*, and *shoulder blade.* SpO_2_ averaging time was set to 6 s. The sensor temperature was set to 44 °C for most sensors. For increasing statistical variety, several measurements were done at 37 °C and 41 °C.

### Statistical analysis

Descriptive statistics (mean/standard deviation or median/range, depending on the distribution of the data) are given for demographic data (age, gender, and BMI). Correlation and Bland-Altman analyses were performed in order to determine Pearson’s correlation coefficient (*r*), bias (*d*), and standard deviation (SD). In concordance with the uniformity of data presentation that follows from guideline ISO 80601-2-61:2011, the accuracy root mean square error (*A*_rms_) was calculated with limits of agreement that did not take repeated measurements into account ($$ \mathrm{Arms}=\sqrt{\frac{\sum \limits_{i=1}^n{\left({\hat{y}}_i-{y}_i\right)}^2}{n}}=\sqrt{d^2+{\mathrm{SD}}^2} $$$$ \mathrm{Arms}=\sqrt{\frac{\sum \limits_{\mathrm{i}=1}^{\mathrm{n}}{\left({\hat{\mathrm{y}}}_{\mathrm{i}}-{\mathrm{y}}_{\mathrm{i}}\right)}^2}{\mathrm{n}}}=\sqrt{{\mathrm{d}}^2+{\mathrm{SD}}^2} $$). In this formula, $$ {\hat{y}}_i $$ is the SpO_2_ value for iteration number *i*, *y*_*i*_ is the measured SaO_2_ value for the iteration number *i*, *n* is the number of samples, and *d* is the bias. The presented limits of agreement and the between-subject variance were calculated according to the methods of repeated measurements as described by Bland and Altman [31].

## Results

### In vitro accuracy and drift of tcPO_2_ and tcPCO_2_

**Fig. 2:**
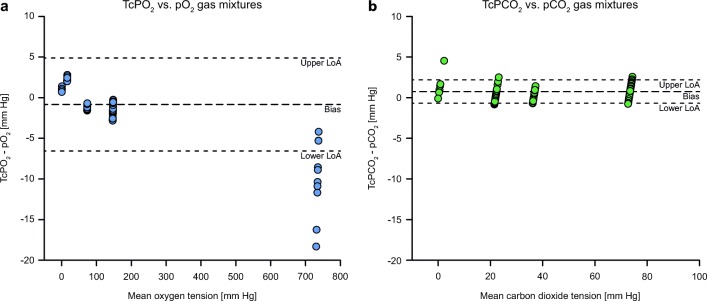
Bland-Altman plots of the agreement of in vitro tcPO2 (a) and tcPCO2 (b) measurements with calibration gas mixtures. The accuracy, bias, and limits of agreement (LoA) for each measurement are shown in Table 1.

A total of 17 tcPCO_2_ and 18 tcPO_2_ data points were collected with each of the 10 sensors. The number of available data points and the correlation and Bland-Altman analyses of the tcPO_2_ and tcPCO_2_ data compared with the gas O_2_ and CO_2_ partial pressures are shown in Fig. 2 and summarized in Table 1. At oxygen tensions of over 700 mm Hg, agreement of tcPO_2_ with the reference gas has decreased, underestimating the pO_2_. Measurement drift over different intervals shows a very small overall O_2_ drift (Table 2). Drift of tcPCO_2_ is notably highest during the first hour, tcPO_2_ drift is not equally affected.

**Table 1:** In vitro accuracy of tcPO_2_ (0–100%) and tcPCO_2_ (0–10%) measurements

Measurement	Data points (*n*)	Accuracy (mm Hg)	Bias (mm Hg)	Limits of agreement (mm Hg)		*r*
		*A*_rms_		Lower	Upper	
tcPO_2_	180	3.0 (2.9)	− 0.8	− 6.6	4.9	0.999
tcPCO_2_	170	1.1 (0.7)	0.8	− 0.7	2.2	0.999

Values measured with the OxiVenT™ Sensor and compared with calibration gas mixtures

**Table 2:** Data on drift of tcPO_2_ (24-h calibration interval) and tcPCO_2_ (12-h calibration interval)

	Total drift during calibration interval (12 h/24 h) (%)	Drift during first hour of calibration interval (%/h)	Drift during last hour of calibration interval (%/h)
tcPO_2_	0.30 (0.63)	0.14 (0.28)	0.03 (0.21)
tcPCO_2_	− 2.53 (1.04)	0.49 (0.28)	0.18 (0.09)

Data is shown as mean (SD)

### Clinical use of tcPO_2_ and tcPCO_2_

**Fig. 3:**
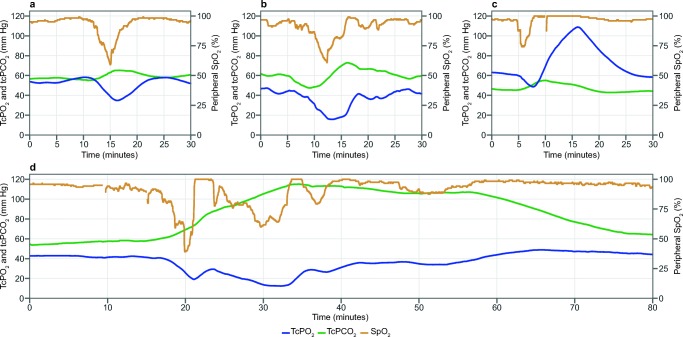
Clinical examples of tcPO_2_ and tcPCO_2_ measured in preterm neonates with the OxiVenT™ Sensor during relevant events, supplemented with standard of care peripherally measured transmission pulse oximetry. These examples show the following events: (a) Very preterm neonate, born at a gestational age (GA) of 28 weeks and with a birth weight (BW) of 1200 g. Drop in oxygen saturation to 56% SpO_2_ due to retention of sputum, following by suctioning, accompanied by a transient rise of tcPCO_2_ and decrease of tcPO_2_ down to 35 mm Hg. (b) Extreme preterm neonate, GA 27 weeks, BW 800 g. Capillary blood sampling at an extremity, leading to agitation and crying with a consequential drop in oxygen saturation to 55% and tcPO_2_ to 16 mm Hg. Noteworthy is the temporary drop in tcPCO_2_ due to crying, followed by a rise due to a decline in respiratory effort. The patient’s lungs were recruited due to clinical indications of bronchospasms. The FiO_2_ was increased from 0.21 to 0.40 during this process. (c) Late preterm neonate, GA 36 weeks, BW 2500 g. Short period of bradycardia which was followed by a drop in oxygen saturation. As a clinical intervention, the FiO_2_ was increased from 0.21 to 0.39 for 4 min, leading to a period of hyperoxia up to 109 mm Hg that was undetected by pulse oximetry. (d) Extreme preterm neonate, GA 24 weeks, BW 700 g. During nursing with patient repositioning multiple episodes of bradycardia down to 50 heart beats per minute, with drops in SpO_2_ down to 40% and slow recovery. The decline in respiratory effort and slow recovery are reflected by the clear and persistent elevation of CO_2_ levels

Four examples of clinical events were selected from patient files, are shown in Fig. 3, and include tcPO_2_ and tcPCO_2_ data, as well as the SpO_2_ data obtained from standard of care pulse oximetry. These examples contain both cardiorespiratory patient events and related clinical interventions. A tcPO_2_ response time of approximately 2 min longer when compared with SpO_2_ and a consequential dampening effect can be observed.

### Validation of SpO_2_ in healthy volunteers

**Fig. 4:**
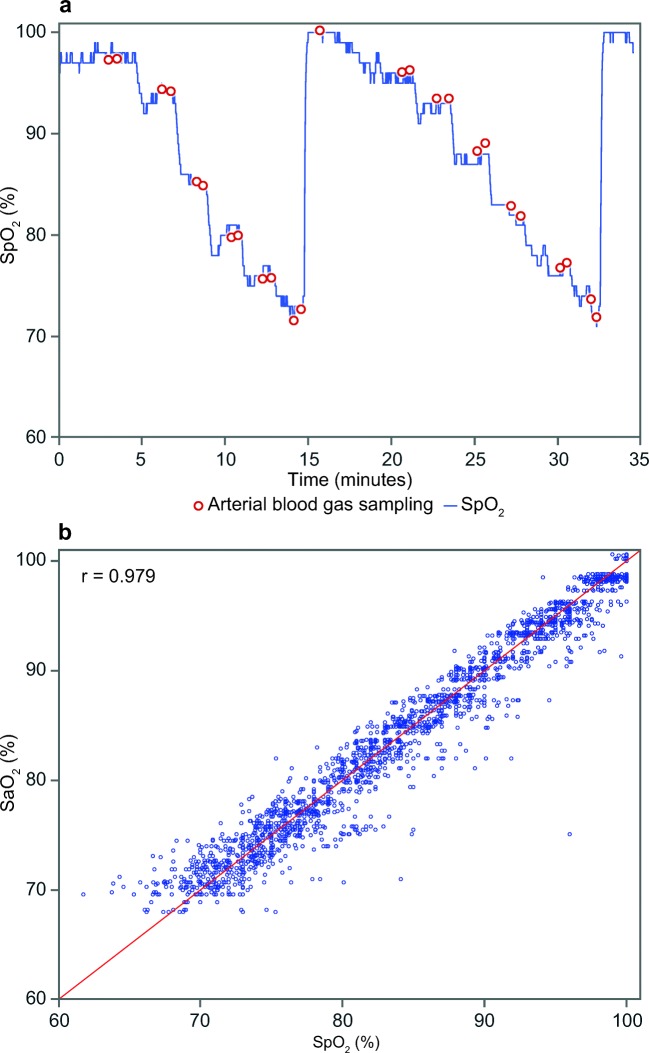
(a) Single-patient example of the controlled desaturation protocol in which a stepwise decrease in SpO_2_ is achieved by controlled lowering of the FiO_2_. Dots indicate the moments when an arterial blood sample was taken during a relative plateau phase. (b )Correlation plot (*r* = 0.979) of SaO_2_ in % from all arterial blood samples and SpO_2_ in % measured with the OxiVenT™ Sensor at the moment of blood sampling (*n* = 2244)

A total of 12 healthy volunteers participated in the study. The study demographics are shown in Table 3. At each step of the test protocol, two blood samples were drawn, of which a single-patient example is shown in Fig. 4(a). This resulted in a total of 2244 SaO_2_-SpO_2_ data pairs. The median of all measured SaO_2_ values is 84.8% (IQR 76.1–93.4%, range 68.0–100.6%). The correlation plot of the SaO_2_ values with the corresponding SpO_2_ measurements obtained with the OxiVenT™ Sensor at all five measurement sites is shown in Fig. 4(b). The accuracy and agreement analyses for the separate measuring sites show the narrowest limits of agreement when measuring at the forehead and cheek, with the highest accuracy when measured at the forehead (Table 4).

**Table 3:** Volunteers characteristics

No. of volunteers	12
Age (years)	25 (23–34)
Male/female	7/5
Skin type	
- Dark	2
- Medium	5
- Light	5

Values are listed as median (range), where applicable

**Table 4:** Agreement between SpO_2_ and SaO_2_ at different measurement sites

Application site	SaO_2_-SpO_2_ pairs	Accuracy (%)	Bias (%)	Limits of agreement (%)		*r*
		*A*_rms_		Lower	Upper	
Earlobe	451	2.44	1.16	− 3.17	5.49	0.973
Forehead	526	1.35	− 0.29	− 2.89	2.31	0.990
Cheek	476	1.29	0.56	− 1.75	2.86	0.992
Upper arm	415	2.41	0.38	− 4.39	5.15	0.971
Shoulder blade	376	2.13	− 1.34	− 4.64	1.96	0.989
Overall	2244	2.09	0.13	− 3.17	3.98	0.979

Pooled SaO_2_-SpO_2_ A_rms_ (not corrected for repeated measurements), bias, limits of agreement (corrected for repeated measurements), and Pearson’s correlation coefficient (not corrected for repeated measurements) per application site

## Discussion

With these studies, we present data on the OxiVenT™ Sensor, the first combined tcPO_2_, tcPCO_2_, and SpO_2_ transcutaneous sensor incorporating an optical tcPO_2_ measurement that is designed to eliminate measurement drift. The in vitro results confirm a good tcPO_2_ accuracy and negligible overall measurement drift. Decreased tcPO_2_ accuracy and precision can be observed at very high oxygen tensions, together with underestimation of PaO_2_. This is most likely a consequence of the abundance of oxygen, leading to a short fluorescence decay time in combination with a high intensity. However, these supraphysiological levels are not likely to be clinically relevant. TcPCO_2_ accuracy and drift are on par with previous sensor generations [32]. TcPCO_2_ drift is highest during the first hour of measurement, possibly due to equilibration effects. TcPO_2_ drift does not seem to be equally affected, providing a more consistently accurate measurement from onset. Furthermore, SpO_2_ shows excellent correlation and agreement with SaO_2_ values in adult volunteers, particularly when measuring at the forehead or cheek. Although transcutaneous blood gas measurements have retained their place in the clinic after the introduction of pulse oximetry, the technique has remained laborious [33–35]. When measurements are considered to be in disagreement with arterial values, they require training to be able to distinguish technical failure or measurement drift from patient factors influencing the measurement. As a consequence, transcutaneous monitoring is most often used when the required dedicated attention is outweighed by the advantages, such as in neonatal intensive care units or sleep laboratories. The logical innovation in transcutaneous blood gas monitoring is consequently the introduction of drift-free measurement techniques, making transcutaneous monitoring more accurate and easy to use. In the investigated OxiVenT™ Sensor, an optical tcPO_2_ measurement has been implemented for this purpose. The main patient-related limitation of transcutaneous tcPO_2_ and tcPCO_2_ measurements is inaccuracy due to the influence of skin thickness and microcirculatory impairment on the diffusion of blood gases [2, 18, 36]. TcPO_2_ accuracy is known to suffer more from these influences than tcPCO_2_ accuracy due to the higher skin diffusion resistance to oxygen [4], leading to wide limits of agreement in clinical studies on tcPO_2_ [11, 37]. In addition, the traditional electrochemical tcPO_2_ sensors contained Clark-type electrodes, which consume oxygen as part of the measurement [2, 6]. The implementation of an optical measurement technique for tcPO_2_ therefore potentially has a greater measurement technique–related impact on accuracy for than it would have for tcPCO_2_. Clinical measurements of tcPO_2_ and tcPCO_2_ in the Neonatal Intensive Care Unit suggest good usability and response to clinical events. The relatively long tcPO_2_ response time makes it unsuitable for detecting apneic episodes and oxygenation dips. In adults, the inability to measure tcPO_2_ values that mirror PaO_2_ values limits the use in the adult population to oxygen trend monitoring. However, the improved reliability of the tcPO_2_ trend could clinically have a greater impact than improved agreement with blood gas samples. Data on the user preference of using either absolute values or trends is however limited and specific for patient populations. With the new OxiVenT™ Sensor, the potential of optical techniques has been demonstrated. In clinical use, this combined sensor will however still require frequent calibration of the electrochemical tcPCO_2_ measurement, negating the potential benefit on calibration strain for both patients and personnel. Although this study provides useful information on the technical performance of this new combined sensor, clinical validation is needed to evaluate its impact and limitations.

## Conclusion

Our results show the successful integration of a new optical oxygen measuring technique in a non-invasive, combined tcPO_2_, tcPCO_2_, and SpO_2_ sensor. In vitro tcPCO_2_ measurement performance is unchanged when compared with literature on previous sensor generations. Reflectance pulse oximetry correlates well in a study on healthy volunteers. The new optical tcPO_2_ measurement is virtually drift-free in vitro. Despite showing good usability in clinical examples, the clinical benefit needs to be proven. Additionally, clinical data is needed to validate this sensor to arterial blood samples in specific patient populations.
